# Self-Organized Criticality, Plasticity and Sensorimotor Coupling. Explorations with a Neurorobotic Model in a Behavioural Preference Task

**DOI:** 10.1371/journal.pone.0117465

**Published:** 2015-02-23

**Authors:** Miguel Aguilera, Xabier E. Barandiaran, Manuel G. Bedia, Francisco Seron

**Affiliations:** 1 Dept. of Computer Science and Engineering Systems, Universidad de Zaragoza, Zaragoza, Spain; 2 Department of Philosophy, University School of Social Work, UPV/EHU University of the Basque Country, Vitoria-Gasteiz, Spain; 3 Department of Logic and Philosophy of Science, IAS-Research Center for Life, Mind, and Society, UPV/EHU University of the Basque Country, Donostia-San Sebastián, Spain; National Scientific and Technical Research Council (CONICET), ARGENTINA

## Abstract

During the last two decades, analysis of 1/ƒ noise in cognitive science has led to a considerable progress in the way we understand the organization of our mental life. However, there is still a lack of specific models providing explanations of how 1/ƒ noise is generated in coupled brain-body-environment systems, since existing models and experiments typically target either externally observable behaviour or isolated neuronal systems but do not address the interplay between neuronal mechanisms and sensorimotor dynamics. We present a conceptual model of a minimal neurorobotic agent solving a behavioural task that makes it possible to relate mechanistic (neurodynamic) and behavioural levels of description. The model consists of a simulated robot controlled by a network of Kuramoto oscillators with homeostatic plasticity and the ability to develop behavioural preferences mediated by sensorimotor patterns. With only three oscillators, this simple model displays self-organized criticality in the form of robust 1/ƒ noise and a wide multifractal spectrum. We show that the emergence of self-organized criticality and 1/ƒ noise in our model is the result of three simultaneous conditions: a) non-linear interaction dynamics capable of generating stable collective patterns, b) internal plastic mechanisms modulating the sensorimotor flows, and c) strong sensorimotor coupling with the environment that induces transient metastable neurodynamic regimes. We carry out a number of experiments to show that both synaptic plasticity and strong sensorimotor coupling play a necessary role, as constituents of self-organized criticality, in the generation of 1/ƒ noise. The experiments also shown to be useful to test the robustness of 1/ƒ scaling comparing the results of different techniques. We finally discuss the role of conceptual models as mediators between nomothetic and mechanistic models and how they can inform future experimental research where self-organized critically includes sensorimotor coupling among the essential interaction-dominant process giving rise to 1/ƒ noise.

## Introduction

The tools and concepts of complex systems research (often forged in the realm of physics) are increasingly permeating cognitive science and the way we understand the mind and ourselves. Since early cybernetics [1] and system’s theory [2, 3], what might currently be called the complex systems approach to cognition, has put the emphasis on self-organization, the irreducible non-linearity of cognitive processes and circular causality [4–6]. This approach contrasts with some of the classical assumptions behind the computational theory of the mind: modularity, functional decomposition, perception-action dichotomy, agent environment ‘decoupling’ (sharp analytic separation between the dynamics of the agent and the environment), etc. The complex systems approach to cognitive science seeks to move beyond the formalism of computational theory and information exchange and to place cognition within the broader formalism of dynamical systems [7–9].

Paradigmatic examples of the conceptual tools of the complex systems approach, with applications in several domains (in particular in cognitive science and neuroscience), are the concepts of self-organized criticality (SOC hereafter) and 1/*f* noise (also known as scale-free or pink noise) [10, 11]. The concept of SOC was initially proposed by Bak, Tang and Wiesenfeld [10] to define certain classes of dynamical systems which have a critical point as an attractor, displaying critical behaviour without any significant ‘tuning’ of the system from the outside. Critical systems have very interesting properties, the most characteristic of which is the lack of a dominant scale of activity. They show complex dynamical responses and their statistical properties have to be described by power laws. Thus, critical systems typically display temporal and spatial scale invariance in the form of fractals and 1/*f* noise, reflecting the process of propagation of long-range interactions based on local effects. For the complex systems approach to cognitive science SOC is appealing because it allows us to imagine systems that are able to self-regulate coordinated behaviours at different scales in a distributed manner and without a central controller.

### SOC in Neuroscience, Cognitive Science and Psychology

It has been shown that the brain is in a continuous state of SOC. Experimental evidence and artificial neural network models support this hypothesis. Criticality in the brain is suggested by power law scaling in degree distributions of functional brain networks or avalanche size distributions [12]. Long-range correlations in the form of 1/*f* patterns have been extensively found in EEG/MEG measurements of brain activity in alpha, mu, and beta oscillations [13]. Scale-free neocortical dynamics has also been ascertained by Freeman [14] in EEG measurements in rabbits. In addition, there are many instances of critically self-organized behaviour in artificial network models, which provide some interesting insights [15]. For example, it has been shown that a broad (robust) critical regime is favoured by the presence of strong synaptic interaction and high synaptic time-dependent plasticity [16].

SOC and 1/*f* have also been extensively found in cognitive science and psychology. For example, 1/*f* noise is present in time series showing how performance fluctuates over time [17]. More recently, Van Orden, Holden and Turvey [8, 18] used 1/*f* noise measurements in different tasks to gather evidence to defend the idea that certain systems are not modular and decomposable but are ‘softly assembled’ systems sustained by *interaction-dominant dynamics* (IDD hereafter) as opposed to *component-dominant dynamics* [8]. That is, IDD systems do not consist of additive interactions of their components, but of multiplicative interactions that imply coordination between the different timescales in the system. Silberstein and Chemero go further and suggest that 1/*f* measurements can be an indicator of *extended cognitive systems*, in which strongly nonlinear and softly assembled interactions allow the emergence of a brain-body-environment system of nested self-organization [19]. These hypotheses are inspired by measurements of 1/*f* noise in a simple human-tool interaction with and without perturbations in the interface between *human and tool* [20]. In this context, ‘extended’ means that it is impossible to determine the contribution of every individual part to the behaviour of the system as a whole. Instead, under this framework, these authors attempt to characterize holistic integrated behaviours that can account for locally irreducible properties at the whole agent level (what some philosophers call the ‘personal level’): intentionality, free will, agency, and so on [21]. Self-organized criticality thus becomes an interesting candidate for explaining the holistic and integrated nature of mental life; and its 1/*f* noise manifestation a signature or quantitative indicator of this holistic integration.

One of the most promising approaches to the ‘unity’ or ‘integratedness’ of mental life was suggested by Varela [22], proposing that mental-cognitive states are mediated by a specific cell assembly (the term was originally coined by Hebb [23]) that emerges through transient phase locking of distributed neural regions. Every cognitive act corresponds to the emergence of one dominant assembly which incorporates or discards cortical and subcortical regions into a unified and transitory whole. More recently, Tononi and Edelman [24] have used the term *dynamic core* to describe this process, emphasizing both its integration and its ability to generate an extraordinary variety of complex patterns. Ever since, the notion of a dynamic core has become relatively widespread in large-scale neuroscience studies [25–29]. SOC has been suggested as a candidate for characterizing this state between integration and segregation, allowing the system to remain in a self-organized state that is at the same time stable (or metastable) while also extremely sensitive to small microscopic perturbations, and with the capacity to develop long-range correlations at all the scales of the system without requiring a costly fine tuning of local parameters [30]. In turn, the presence of 1/*f* noise has been proposed as an indicator of the long-term evolution of a dynamic core in EGG measurements [31].

### The Controversy over the Explanatory Capacity of 1/*f* Noise Measurements

Despite the enthusiasm brought by the widespread finding of 1/*f* noise, some authors remain cautious about its specific interpretation. Van Orden *et al*. claim that ubiquitous pink noise is not sufficient evidence of self-organized criticality but rather a necessary consequence [8, p. 343]. They argue that while it is possible to interpret 1/*f* noise in some contexts as a signature of a softly assembled system (self-sustained by IDD and SOC) this is not always necessarily the case. Moreover, 1/*f* is not a unique and exclusive property of SOC systems (see [32, 33]) since it can be displayed by a linear superposition of random components acting on multiple time scales [34]. To avoid the uncertainty about the true origin of 1/*f* noise some authors have suggested complementing it with a measure of multifractality as a quantitative indicator of interaction-dominant dynamics, providing a means to ensure the nonlinear nature of the ongoing interactions that build the self-organized process [35].

But the feasibility of different types of indicators or measurements is not the only problem. At the theoretical level there still remains considerable controversy as to when a system can be considered to display SOC properties (e.g. [11]). Bak, Tang, and Wiesenfeld’s original use of the term SOC was based on a loose *phenomenological definition*: a system that exhibits power laws or 1/*f* fluctuations without any apparent tuning is said to exhibit self-organized criticality [10]. However, sometimes 1/*f* patterns found in connection with the search for SOC extend only over a narrow frequency interval, and sometimes in genuine critical systems not all observables will show 1/*f* behaviour. Some authors have pointed out the limitations of mere descriptive or phenomenological approaches in which 1/*f* is identified and explained just by verbal descriptions and metaphors of SOC without specifying concrete models or mechanistic requirements to identify a system as operating under SOC [33].

*Generative or mechanistic definitions* have also been provided where the observed phenomena are systematically related to the underlying mechanisms or organizational principles. According to Jensen [11], SOC systems are slowly driven, interaction-dominated threshold systems: many degrees of freedom interact, and the dynamics of the system is dominated by the mutual interaction between those degrees of freedom. Fractal scaling arises from the fact that the external driving of the system is much slower than the internal relaxation processes, and the existence of thresholds and metastability in the system ensures that it can be driven by the interactions between its different components. Jensen’s definition of SOC allows us to go further in our understanding of the phenomenon, modelling and testing concrete hypotheses about critically self-organized behaviour. An example, pointed out by Jensen, is the difference between sand piles (which exhibit avalanches with periodic behaviour) and rice piles (which display a broad distribution of avalanche sizes characteristic of SOC) [11, p. 126]. In a sandpile, the kinetic energy (gravitational pull) of individual grains dominates the friction between grains, whereas in a ricepile the motion of a single grain is easily stopped by intergrain friction forces. Intergrain friction produces a threshold (‘local rigidity’) that allows a huge number of possible metastable states. Jensen suggests that the existence of thresholds is a necessary condition for the interaction-dominant dynamics necessary for SOC. This approach allows us to generate hypotheses that can be tested by mathematical modelling and experimental results.

### Filling the Gap: Exploring the Role of Active Sensorimotor Modulation in the Generation of 1/*f* Noise

Whereas evidence for the presence of 1/*f* noise in neural dynamics and in certain experimental psychology tasks is apparent (mostly behavioural data in the latter case), there is very little understanding of how both neurodynamic and behavioural levels relate to each other and to 1/*f* noise. Moreover, most models proposing generative mechanisms for the emergence of 1/*f* noise are focused on either a behavioural [36] or a neural level [16, 37].

Although insightful, these models fall short of providing in-depth explanations about the nature of neural and behavioural organization in embodied subjects in a softly assembled brain-body-environment system, because in such models the systems are never shown to operate embedded in a specific environment.

In this paper, we aim to address this gap by exploring the role of active sensorimotor modulation in the generation of 1/*f* noise and SOC, i.e., how neural, embodied and environmental dynamics interact together to generate critically self-organized dynamics. Since previous approaches (detailed mechanistic models or conceptual nomothetic analysis) have not yet proven to be especially fruitful for addressing the interaction between neural and behavioural scales, we propose an approach in which we exploit a minimal conceptual robotic model of brain-body-environment interaction. Following the theoretical modelling tradition of evolutionary robotics, which has for a long time contributed specific models for abstract concepts [38, 39], we present a conceptual model depicting a simulated robot in a behavioural preference task. A conceptual model differs from detailed mechanistic models in the sense that it does not target a specific task or specific empirical data, but rather implements known principles and mechanisms and generates qualitatively significant results that make it possible to advance hypotheses about the nature of explanations, to make explicit complex and non-intuitive relationships between levels of description, to provide proofs of concepts, etc. [40]. We do not aim to obtain universal rules for the relation between neural and behavioural levels of description, but we hope to clarify to some extent what kind of mechanisms and principles (sensorimotor coupling, plasticity, etc.) may underlie the generation of 1/*f* noise and SOC, at least in some cases.

In the next section we describe our model, its neural controller (a Kuramoto oscillatory network with a relational homeostatic mechanism), embodiment and environment, together with the details of the artificial evolutionary optimization process used to make it solve a behavioural preference task. We then analyze the behavioural dynamics of the agent and its relation to the presence of synaptic plasticity and sensorimotor coupling. We analyze 1/*f* patterns in our agents for different conditions of internal plasticity and absence of sensorimotor interaction. Finally, we discuss the results obtained and their impact on the current state of complex systems approaches to cognition. We conclude with some remarks about the model presented and the proposed modelling strategy.

## Model

We follow the work of Di Paolo and colleagues on autonomy in evolutionary robotics [41–43], extending previous models of homeostatic adaptation. Unlike previous models, where continuous time recurrent neural networks (CTRNN) were used as robot controllers, this model is implemented in a simulated mobile agent with a plastic Kuramoto network as a neural controller, with an additional loop of homeostatic regulation in which the homeostatic zone favours some preferred phase relations between the network’s oscillators. The simulated robot is presented with two lights of different colours that are perceived by two different pairs of sensors, and evolved using a genetic algorithm to develop switching robust preferences to the two types of lights. All parameters (except when specified otherwise) are determined genetically within the indicated range.

### Kuramoto Oscillator Networks with Relational Homeostatic Plasticity

A fully connected Kuramoto network [44] with three oscillators is used as the agent’s controller. The evolution of the state of each oscillator is defined by:

$$\begin{array}{c} {\dot{\theta }}_{i}=\omega_{i}+I_{i}+\sum_{j=1}^{N}K_{ij}\cdot sin\left(\theta_{j}-\theta_{i}\right) \end{array}$$ (1)

where *θ*_*i*_ represents the phase of oscillator *i*, *ω*_*i*_ is its natural frequency (range [0, 5]), *K*_*ij*_ is the strength of the coupling between the oscillator *i* and the oscillator *j* and *I*_*i*_ represents the sensory inputs, which are given only to sensory neuronal oscillators 1 and 2.

This model is not intended to represent the activity of individual neurons but, more generally, to capture the dynamics of neural oscillations at a mesoscopic level. Integration mechanisms in the brain are hypothesized to be based on phase synchronization processes between neuronal groups [29], thus we aim to represent the large-scale synchronization of brain regions that are far apart in the brain.

In order to measure *global* levels of synchronization we have extended the Kuramoto model to include the relations of one oscillator with respect to its neighbours. This new parameter *ϕ* is computed by the phase difference of one oscillator with respect to the sum of the oscillator cluster connected to it weighted by the strength of their connections. It is represented by:

$$\begin{array}{c} \phi_{i}=∠\left(\sum_{j=1}^{N}K_{ij}\cdot e^{i(\theta_{j}-\theta_{i})}\right) \end{array}$$ (2)

where ∠ denotes the phase of the result of the summatory and i is the imaginary unit. Here *ϕ*_*i*_ represents the weighted phase relation between oscillator *i* respect to the other oscillators which it is connected.

**Fig 1:**
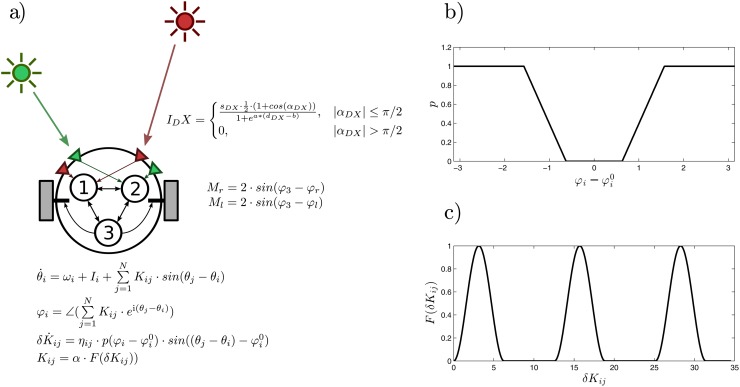
The robotic agent with three plastic oscillatory units. a) Scheme of the agent, the environment, sensors and motors, and the neural controller b) Plastic function $p(\phi_{i}-\phi_{i}^{0})$, in which plasticity depends on the difference between the weighted phase relation *ϕ*_*i*_ of the neural oscillator *i* with respect to other oscillators and the preferred weighted phase relation $\phi_{i}^{0}$ c) Mapping function *F*(*δK*_*ij*_) which transforms weight values *δK*_*ij*_ into the actual value of coupling strengths between oscillators *K*_*ij*_.

A plastic mechanism is added to the Kuramoto network to homeostatically modify the weights of the connections between neurons. Homeostatic regulation is defined by a stepwise function that determines synaptic plasticity as a function of the phase relations *ϕ* between an oscillator and its connected cluster, $p(\phi_{i}-\phi_{i}^{0})$, where the homeostatic region is located around a preferred phase relation $\phi_{i}^{0}$ (range $\left[\frac{-\pi }{2},\frac{\pi }{2}\right]$). The function *p*(*x*) (Fig. 1.b) is defined by two activation thresholds *H*_1_ and *H*_2_ (range $\left[0,\frac{\pi }{5}\right]$). The value of *p*(*x*) is 0 when *x* < *H*_1_, it increases linearly while *H*_1_ < *x* < *H*_2_ and it is equal to 1 when *H*_2_ < *x*. We have arbitrarily set the values of *H*_1_ = 0.2*π* and *H*_2_ = 0.2*π*. That is, neural oscillations connect to each other in such a way that they can show preferred phase relations with other oscillating clusters. This function determines the level of plastic change for all incoming weights. Within the homeostatic region the value of the plastic function is 0, which means that no plastic changes take place while the oscillator stands within the boundaries of its preferred phase relation with the cluster to which it is connected. Though the implemented homeostatic mechanism may be arbitrary, there is evidence of how brain networks adjust their temporal relations with great precision by plastic mechanisms [45].

Changes in the value of the incoming weights of oscillator *i* depend on the local plasticity of oscillator *i*, $p(\phi_{i}-\phi_{i}^{0})$, multiplied by an extra term which determines the level of synchronization between oscillator *i* and each incoming oscillator *j*, the plasticity being smaller for oscillators with higher levels of phase synchronization:

$$\begin{array}{c} \delta {\dot{K}}_{ij}=\eta_{ij}\cdot p\left(\phi_{i}-\phi_{i}^{0}\right)\cdot sin\left(\left(\theta_{j}-\theta_{i}\right)-\phi_{i}^{0}\right) \end{array}$$ (3)

where *δK*_*ij*_ are the connection weights which are initialized randomly at the beginning of each trial and, *η*_*ij*_ is the rate of change (range [0, 0.9]) of each connection.

Finally, the actual connection strengths are mapped by a modified sinusoidal function of the weights *δK*_*ij*_. A similar strategy was already used in [46] in homeostatic neural networks, instead of using a Hebbian-like rule, to avoid saturation of synaptic strengths. This mechanism makes sure that there is enough variability for plastic reconfigurations in order to explore the whole space of connection weights. In our case, we have modified the sinusoidal mapping multiplying it by a positive square wave with half its frequency in order to assure the possibility of total disconnection between oscillators. Thus, when plastic changes take place, connection strengths follow a continuous non-monotonic function *K*_*ij*_ = *α* ⋅ *F*(*δK*_*ij*_) (see Fig. 1.c) capable of exploring the full configuration space, where *α* is a constant (range [0, 5]) that regulates how strong the oscillator coupling can be.

In a nutshell, this minimal model works under the assumption that large-scale neural oscillatory components try to maintain an invariant preferred phase relation with respect to other oscillatory components by means of plastically regulating the strength of their connectivity.

### Minimal Robotic Embodiment. Behavioural Preference Task

The agent is modelled as a simulated wheeled robot with a circular body of radius 4 and two diametrically opposed motors (Fig. 1.a). The motors can drive the agent forward and backwards. We assume that the agent’s mass is small enough to be neglected (in order to avoid inertial resistance), so we can describe the speed of the agent as follows: the translational speed of the robot is calculated as the vectorial average of the motor velocities, and the angular speed as the difference of the motor velocities divided by the body diameter. The motor outputs are calculated from the phase relation *ϕ* of the effector oscillator (oscillator 3). The speed of the motor is obtained by multiplying the motor output by a gain parameter of value 2:

$$\begin{array}{c} \begin{array}{c} M_{r}=2\cdot sin\left(\phi_{3}-\phi_{r}\right)\\ M_{l}=2\cdot sin\left(\phi_{3}-\phi_{l}\right) \end{array} \end{array}$$ (4)

where *ϕ*_*r*_ and *ϕ*_*l*_ (range [0, 2*π*]) are bias terms which map the motor output into the actual motor activation.

The agent has two pairs of sensors (right and left) for each of the different light sources *A* and *B*. Each sensor points to a direction at *π*/3 radians from the forward direction. Light *A* sensors are connected to oscillator 1 and light *B* sensors are connected to oscillator 2. The activation of the sensor depends on the angle between the sensor and the light, such that the maximal activation happens when the sensor faces the light. The effects of both the angle and the distance on the sensor activation are represented by the following function:

$$\begin{array}{c} I_{DX}=\left\{\begin{array}{cc} \frac{s_{DX}\cdot 0.5\cdot \left(1+cos\left(\alpha_{DX}\right)\right)}{1+e^{a*(d_{DX}-b)}}, & |\alpha_{DX}|\leq \pi /2\\ 0, & |\alpha_{DX}|>\pi /2 \end{array}\right. \end{array}$$ (5)

where *X* can represent either light *A* or *B*, *D* stands for either right or left sensor, *α*_*DX*_ is the angle of sensor *DX* to light *X*, *d*_*DX*_ is the distance between sensor *DX* and light *X*, and *a* and *b* have the arbitrary values of 0.03 and 100 respectively. The light intensity received at each sensor is multiplied by a gain parameter *s*_*DX*_ (range [−8, 8]). The resultant value is fed to the corresponding oscillator’s input *I*_*i*_. A full scheme of the robot is represented in Fig. 1.a.

### Artificial Evolution: Fitness Function

A population of 20 agents is evolved using a rank-based genetic algorithm with elitism. Each of the agent parameters *ω*_*i*_, *s*_*DX*_, *α*_*i*_, *η*_*ij*_, *ϕ*_*r*_, *ϕ*_*l*_ and $\phi_{i}^{0}$ is encoded into a 5 bits string representing a real number within the specified range. For each generation, the best 4 agents (20% of the population) pass to the next generation without change. For the remaining slots, pairs of individuals are selected for crossover with a probability proportional to their fitness value, and new individuals are created mixing their genes (bit series) adding a mutation probability of 3% for each gene.

The agents are evaluated for 4 different tasks (as in [42]): a single light A, a single light B, one light A and a blinking light B, one light B and a blinking light A. In the two first tasks, only one light (either A or B) is present, and the agent gains fitness by approaching the light. In the two latter tasks, two lights are presented (one of type A and one of type B) and one of them is blinking. The agent gains fitness by approaching the non-blinking light. The blinking light emits light only with a probability of 0.15 for each time step. The objective of this is to create a ‘dummy’ that encourages the agent to learn to ignore one of the lights while approaching the other. The lights appear at a random distance, [100, 150]. When two lights are present, they appear, from the agent’s point of view, with a random separation within the range [*π*/2, 3*π*/2]. The length of each trial is 125*s*.

**Fig 2:**
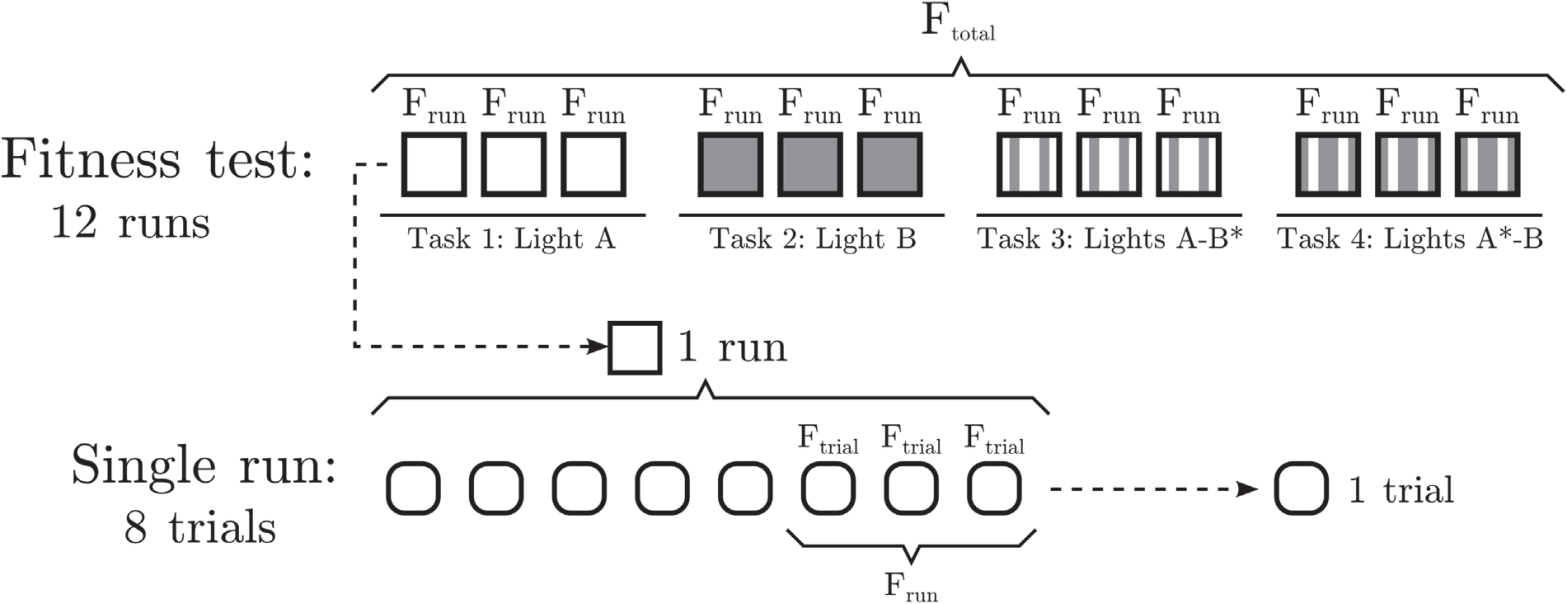
Structure of the task performed by the genetic algorithm. Each agent is tested for 12 different runs (3 for one of the 4 tasks), averaging their fitness values to obtain the total fitness. Each run consists of 8 different trials. For each trial, two lights are presented to an agent during a period of 125*s*. Only the three last trials of a run are taken into account for computing the fitness of the run in order to avoid penalizing slow plasticity.

Each individual agent is tested for 12 independent runs (3 for each of the 4 tasks, see Fig. 2). Each run consists of some trials where a light or a pair of lights are presented to the agents for a fixed time. At the beginning of each run, the synaptic weights *δK*_*ij*_ are reset to initial random values (note that this is different from [42], where weights were reset to a fixed initial value obtained by evolution). Each run consists of 8 trials. For each trial, one or two lights (depending on the task) are presented to the agent for a specified time. After the trial is finished, a new trial begins and two new lights are presented at new positions. Only the last 3 trials of each run are evaluated in order not to penalize slow plastic changes.

Fitness for each trial is calculated in three terms, *F*_*trial*_ = (*F*_*D*_ + *F*_*p*_) ⋅ *F*_*H*_, where *F*_*D*_ values how close to the light the agent has gotten at the end on each trial. For each trial, *F*_*D*_ = 1 − *d*_*f*_/*d*_*i*_, where *d*_*f*_ and *d*_*i*_ respectively correspond to the final and initial distances to the target light. *F*_*p*_ indicates the proportion of time that the agent spends within a distance of less than 4 times its body radius (i.e. a distance of 16) to the target light during a trial. Finally, *F*_*H*_ represents the proportion of homeostasis in the system, computing the degree of homeostasis $1-p(\phi_{i}-\phi_{i}^{0})$ (i.e., 1 minus the level of plasticity) for each oscillator and averaging the result over time and over the three oscillators. In this way we select agents that remain as homeostatic as possible. The total fitness is calculated and then averaged over all 12 runs.

The code simulating the behaviour of the agent and the parameters obtained from the genetic algorithm can be accessed from the following repository https://github.com/IsaacLab/HNA-robotic-model/tree/master/minimal-preference-task.

## Results

**Fig 3:**
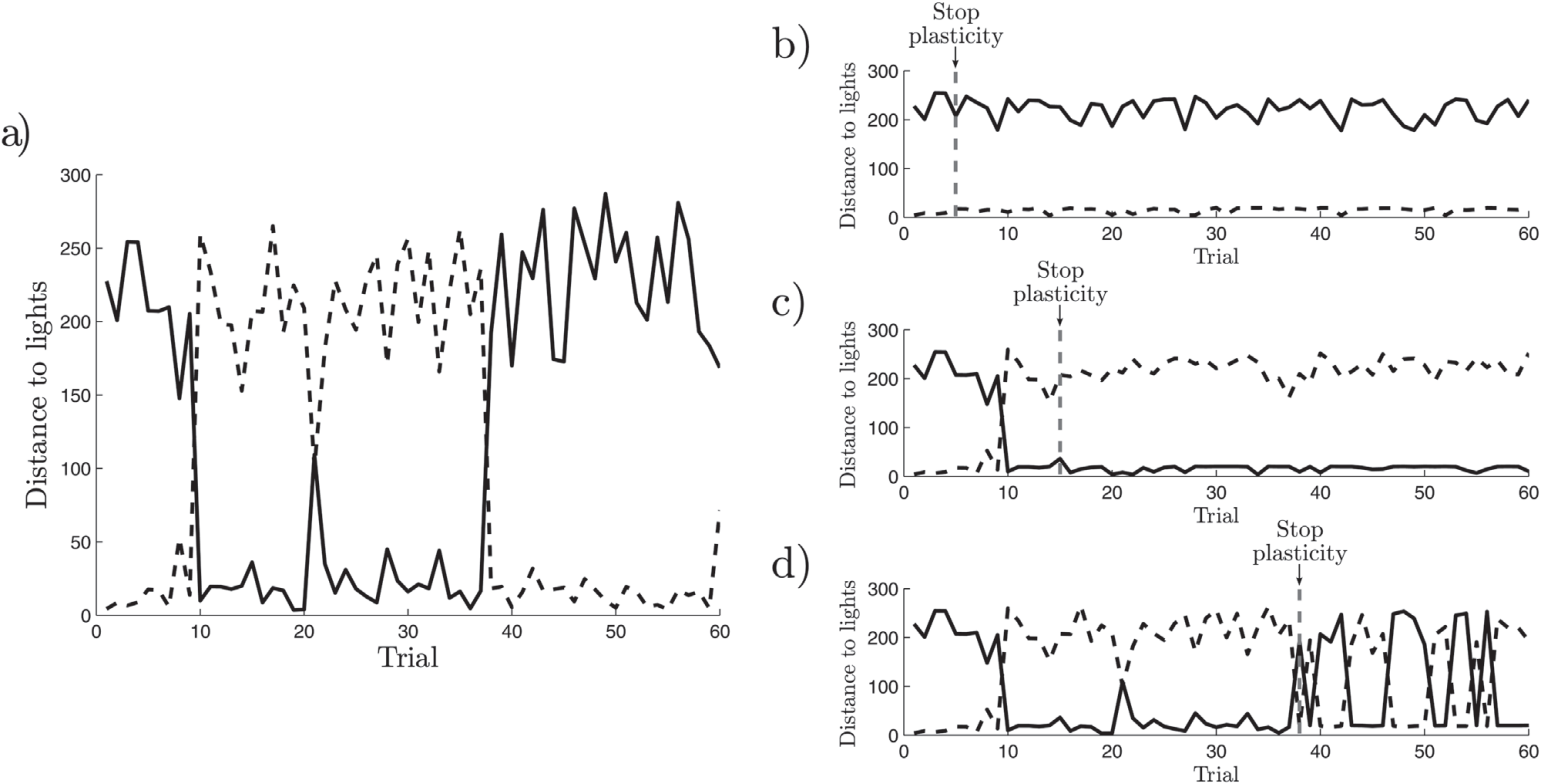
Distance to the two lights at the end of each trial for different agents. A regular agent with synaptic plasticity a), and three agents in which plastic mechanisms have been frozen at different instants; b) in a situation of preference to light A (trial 5); c) in a situation of preference towards light *B* (trial 15), and d) at a moment in which the preference switches from *B* to *A* (trial 38).

After running the genetic algorithm we select the evolved agent from the last generation that obtained the best fitness value. In what follows we analyze the behaviour of this agent but the results can be extended to many other agents that we found to display similar dynamics (i.e. the agent under analysis in this section displays a typical behaviour of this and other evolutionary runs). By analyzing the behaviour of the agent under different conditions, we can test the role played by different aspects of the model in generating the behaviour and the specific effects of such conditions. More specifically we compare the results of agents under the following conditions:

1. A normally functioning agent with both synaptic plasticity and a normal sensorimotor interaction.
2. An agent without synaptic plasticity, where the values of the weights are obtained randomly from a simulation of agent 1 (for the value of the weights at the midpoint of the trials of the simulation represented in Fig. 3).
3. A decoupled agent, that only receives random uncorrelated noise at all its sensors.

We compare these three agents in two ways in the following subsections. The different agents are tested in an environment where the two lights have the same intensity (no dummies). Note that this task (two lights presented with the same intensity) was never carried out during evolution. First, we perform a dynamical analysis of their behaviour and, secondly, we measure the patterns of 1/*f* noise and other fractal indicator of SOC and IDD.

The first step consists of analyzing the behaviour of the agent in a descriptive manner. We present the agent with a series of 60 pairs of lights, and we provide the agent a time of 125*s* to choose and approach one of them. We measure the final distance to both lights at time *t* = 125*s*, obtaining the series represented in Fig. 3.a. The agent is able to develop stable preferences towards one of the lights, maintaining it for several trials until the preference is changed. A video of the behaviour of the agent (including plastic mechanisms) can be found at S1 Video.

### Behavioural Analysis

We now perform a dynamic analysis of the behaviour of the agent and its internal plastic controller. This allows us to test some of the hypotheses about the organization of neural activity and obtain some insights into the processes that give rise to behavioural preferences in our agent. First, we analyze the role of synaptic plasticity in the maintenance of preferences over several trials. We then analyze the neural patterns that sustain transient preferences and give rise to new ones.

#### Synaptic Plasticity

We can test the role of plasticity in the emergence of a new preference by stopping plastic mechanisms at a particular point. We have taken the results of the simulation represented in Fig. 3.a, and run the simulation again under exactly the same initial conditions but freezing the plasticity in the midpoint of a particular trial. We observe in Fig. 3.b and 3.c that when plasticity is stopped the preference is *frozen* and the robot always chooses the same light. Thus, we can say that plastic mechanisms mediate *the creation and destruction of behavioural preferences*. These results show how preferences arise from a complex interplay between the agent’s interaction with its environment and the neural mechanisms of synaptic plasticity. That is, preferences emerge and are maintained when a certain plastic configuration (e.g. sensitiveness to light *A* and blindness to light *B*) are met with certain environmental conditions that allow the agent to maintain its configuration (e.g. the stimuli received from the lights do not trigger plastic mechanisms that may destroy the current sensitiveness of the agent).

Moreover, if we try to stop plasticity during a transition trial (precisely at the moment in which the agent is about to switch its preference, as shown in Fig. 3.d), we observe that the agent continuously alternates between the two lights, being unable to hold a specific preferences. When the weights are frozen at transition points (like the agent in Fig. 3.d) the agent seems to go indifferently to either of the two lights. We call these *undecided* agents. However, are these undecided agents really indifferent to which light to go or do they simply have a shorter preference span?

In order to answer this question we simulate different runs of an agent with synaptic plasticity and several agents without synaptic plasticity of the undecided type. We run the simulations for 1000 trials. For each simulation, we create a time series with the result of the 1000 trials, having a value of 1 when the agent goes to light *A*, and −1 when light *B* is chosen. This time series *D*(*n*) represents the *decisions* of the agent, where *n* is the number of the trial.

First, we can analyze whether the sequences of consecutive trials choosing the same light constitute a consistent preference, or if the agent just chooses randomly and the sequences of same-light consecutive trials are merely happy coincidences. We hold the hypothesis that the agent we called ‘undecided’ (whose connection weights are frozen from an instant in which agent was just switching preferences) does not really possess an internal bias for any light source and whose behavioural choice is the mere result of environmental contingencies. As the configuration of the environment at each trial is completely random, the series of decisions *D*(*n*) would be totally uncorrelated if there is not a mechanism that makes subsequent decisions depend on the choice of the agent in this trial. Thus, we can test the existence of consistent preferences simply by computing the autocorrelation of *D*(*n*), obtaining $\gamma (n)=\sum_{\tau =-1000}^{\tau =1000}D(\tau )\cdot D(\tau +n)$.

**Fig 4:**
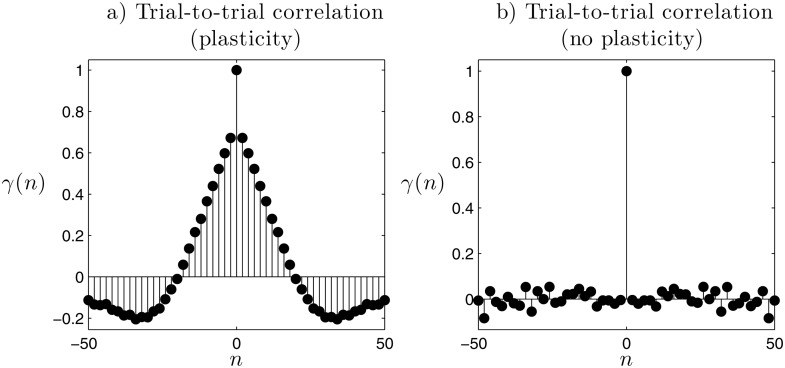
Cross-correlation between the agent’s decisions. We compute the cross-correlation between the time series of the decisions made by the agent at the end of each trial (where 1 means light *A* and −1 light *B*). We observe how consistent trial-to-trial cross-correlations only arise for the agent with synaptic plasticity.

In Fig. 4.a we observe how the agent with plasticity displays strong correlations in its sequence of decisions. The correlation function shows how decisions are positively correlated with the decisions taken in the 20 previous and posterior trials, suggesting that this is the average duration of preferences. In contrast, we see in Fig. 4.b how the ‘undecided’ agent is genuinely undecided and presents no correlations between one decision and the next, meaning that decisions are determined by the randomly generated configuration of the environment. We observe how synaptic plasticity indeed plays a crucial role in the emergence of behavioural preferences, since it is synaptic plasticity a necessary element to produce correlations between one decision and the next.

#### Sensorimotor Interaction

Apart from the role of internal plastic mechanisms, we want to explore role of sensorimotor coupling. Here we test the role of sensorimotor interaction in the emergence of stable neurodynamic patterns in the agent’s oscillatory controller. We do this by comparing the situated agent with a decoupled agent. However, since the decoupled oscillator network alone ends up trapped by an attractor, we stimulated it by feeding the sensors with external signals. First, we stimulated the neural controller with both structured and random signals to find configurations that might produce fractal scaling. Specifically, we tried to feed each sensor with either sinusoidal oscillatory signals or random Gaussian noise. We tested different frequencies for the oscillatory signals and different standard deviations *σ* for the Gaussian noise. We used some of the fractal indicators described in the next section to find which cases present results most closely resembling to 1/*f* noise and we observed that this only occurs in some instances when we feed the network with Gaussian noise signals. Moreover, we observed that only when *σ* was high enough to trigger the plasticity thresholds of the oscillators ($p(\phi_{i}-\phi_{i}^{0})$) were the dynamics of the oscillator network different to white noise. Finally, we found that with a value of *σ* = 1 the decoupled agents presents dynamics resembling 1/*f* noise, so we kept this value for the rest of the experiments.

**Fig 5:**
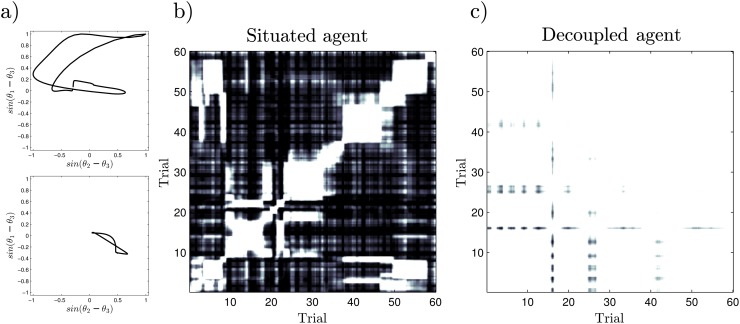
Correlation analysis of the dynamical patterns emerging from the neural controller. We analyze the patterns at different moments for the situated and decoupled agents. In a) we observe different examples of patterns emerging from the situated agent for trials 5 and 15. In b) and c) we observe the correlations between the joint density function of the patterns at different moments of a trial (white for correlation equal to one, black for zero correlation). Clusters of correlated patterns in b) describe the different emergent stable behavioural patterns corresponding to particular preference modes of the situated agent. For the decoupled agent we do not find different clusters of stable behavioural patterns.

Both the situated and the decoupled agents are simulated for a period of time equivalent to 60 trials (7500*s*). For the case of the situated agent the simulation is that represented in Fig. 3.a where the agent is presented with 60 pairs of lights. To analyze the dynamics of the neural controller, we extract segments of the signal taking a sliding window with a width of 125*s* and displacing it along the temporal axis with a slide of 25*s*. Different window sizes were tested with similar results. For each window, we characterize a neurodynamic pattern of behaviour displayed by the internal controller recording the time series within the window of the pairs of variables *sin*(*θ*_1_ − *θ*_3_) and *sin*(*θ*_2_ − *θ*_3_) (note that *θ*_1_ − *θ*_2_ = (*θ*_1_ − *θ*_3_) − (*θ*_2_ − *θ*_3_)). We illustrate two examples of the patterns displayed in trials 5 and 15 for the situated agent in Fig. 5.a. Computing each pattern, we compute the joint density function of the two variables. The density functions are computed using an averaged shifted histogram [47] with 100 bins for each dimension and 8 shifts (although we tested values from 20 to 500 bins with similar results).

After computing the joint probability function of *sin*(*θ*_1_ − *θ*_3_) and *sin*(*θ*_2_ − *θ*_3_) for each window, we have a series of neurodynamic patterns each represented by a matrix of 114×114 elements. To analyze the evolution of different patterns, we compute the correlation of all pairs of matrices over the 60 trials. We can observe the result in Fig. 5.b for the situated agent and Fig. 5.c for the decoupled agent.

Once the correlations of the neurodynamic patterns have been computed we can observe how, for the situated agent, different patterns evolve over time and which instants display similar patterns. This is represented by a temporal clustering in the correlation matrix. For example, we can see in Fig. 5.b how the pattern displayed during trials 3–8 is displayed again during trials 50–58, and comparing with Fig. 3.a we can see how this pattern correspond with periods of time where the agent chooses to go to light *A*. Moreover, we can see how changes of preference coincide with changes of pattern (e.g. in trials 10 and 38). Also, there are situations where the patterns change but where the preference towards one type of light source is maintained (e.g. trials 33, 47). In general we observe well defined and distinguishable patterns, which is consistent with the idea of a dynamic core that sustains autonomous modes of operation. This analysis reveals quite a complex scenario with many different patterns that show different degrees of stability (some are maintained for only one trial while others last for several trials).

On the other hand, when we analyze the decoupled agent (Fig. 5.c), we observe that there no longer any differentiated pattern exist anymore, and all displayed patterns look very much alike. The same happens for different levels of noise activity (different values of *σ*) or when analyzing the patterns using different window sizes. Thus, the emergence of coherent clusters of different behavioural patterns only takes place when neurodynamic activity is modulated by the interaction between agent and environment, where behaviourally distinguishable sensorimotor patterns shape the neural dynamics into different patterns that cannot be sustained by the internal activity of the neural controller alone.

### Self-Organized Criticality: 1/*f* Noise and Multifractality

So far, we have characterized the dynamic formation and dissolution of patterns through the interaction of neurodynamic plastic mechanisms and embodied interaction with the environment. However, is this neural configuration the consequence of a critically self-organized system driven by non-linear interaction-dominant dynamics? In this section we show how fractal and multifractal analyses can provide insights into the types of processes underlying the emergence of behavioural and neurodynamic patterns.

We carefully analyze 1/*f* patterns for the three different agents (situated, decoupled and without synaptic plasticity) to find out if they can be characterized as SOC systems, being especially careful to rule out false positives of 1/*f*-like patterns that are not produced by SOC. We first use two different methods to characterize 1/*f* patterns (fractal and spectral methods). We then measure multifractal exponents for characterizing whether the systems displays IDD. Finally we test if the 1/*f* patterns found are robust to parametrical changes, a property that is definitory of SOC and that allows us to test whether the displayed criticality is truly self-organized.

#### Fractal and Spectral Methods

Since we are analyzing behavioural patters that are maintained over several trials (remember that a trial consists of one presentation of lights and the agent’s ‘decision’ to approach one of the lights), we are interested in measuring long-range correlations at a scale up to many trials. Thus, for the three conditions (situated and plastic, decoupled, and without plasticity) we simulate several runs of an agent facing a series of pairs of lights (again at random distances [100, 150] and random separation angles [*π*/2,3*π*/2]) during 125000*s*. Each time the agent reaches a light (the distance to the light is less than 16 units) or when the agent does not reach the light in a period of 1250*s*, the lights disappear and two new lights appear at random positions. We measure 1/*f* correlations in a signal $\Phi =|H(\frac{1}{3}\sum_{i=1}^{3}\;\sin(\theta_{i}))|$, where *H*(*x*) is the Hilbert transform of signal *x*. Here Φ can be interpreted as the amplitude envelope of the mean activation signal of the oscillatory Kuramoto network. Taking the amplitude envelope of a signal is a widespread practice when analyzing fractal exponents of neural oscillatory signals (e.g. see [48]).

As we saw in the Introduction, simply identifying a linear slope in a logarithmic representation is not enough for characterizing 1/*f* noise. In [18] Van Orden *et al*. propose to use fractal and spectral methods in tandem to avoid the mistake of taking transient correlations for scaling relations. We therefore use two different methods for characterizing 1/*f* dynamics: the discrete Fourier transform (DFT) and detrended fluctuation analysis (DFA). DFT allows us to decompose a signal into its different frequency components. Then, we use the Welch method [49] to estimate the signal’s power spectrum. If the power spectrum has the form of a 1/*f* ^*β*^ function we say that the signal exhibits fractal dynamics. Usually pink or 1/*f* noise is considered to correspond to values of *β* between 0.5 and 1.5. Similarly, values of *β* close to 0 correspond to white noise (uncorrelated processes) and values close to 2 to brown noise (process driven by slow timescales showing short-term predictability). Only processes with *β* around 1 are considered to display SOC [11].

DFA [50] is a method for determining the statistical self-affinity of a signal. In a nutshell, the DFA algorithm integrates the analyzed time series and then divides it into boxes of equal length *n*. For each box and each value of *n*, the least squares line (the trend of the signal within the box) that best fits the data is extracted. For each box of size *n*, the characteristic size of the fluctuation *F*(*n*) is computed as the root mean square deviation between the integrated signal and its trend in each box. This computation is repeated for every value of *n*. Typically, *F*(*n*) increases with *n*. A linear relationship on a log-log plot with slope *α* indicates the presence of fractal scaling in the analyzed signal, where *α* is a generalization of the Hurst exponent, which is related to the scaling in the Power Spectrum of the Fourier analysis being *β* = 2 ⋅ *α* − 1.

DFA has some advantages compared to DFT analysis. While DFT is only well suited for stationary signals, DFA has been reliably used for non-stationary signals. Moreover, results from DFT are often noisy and sometimes not absolutely reliable for determining linear relationships in logarithmic scales (e.g. see [32]). Here we compute the average of the DFTs for different runs.

After generating a series of the amplitude envelope of the mean network activation Φ over 1000 trials for the three different conditions (situated, decoupled and non-plastic), we can apply DFA and DFT algorithms to the signal. We focus our analysis in a temporal scale ranging from 1*s* to 10^5^ *s*.

#### Detrended Fluctuation Analysis

**Fig 6:**
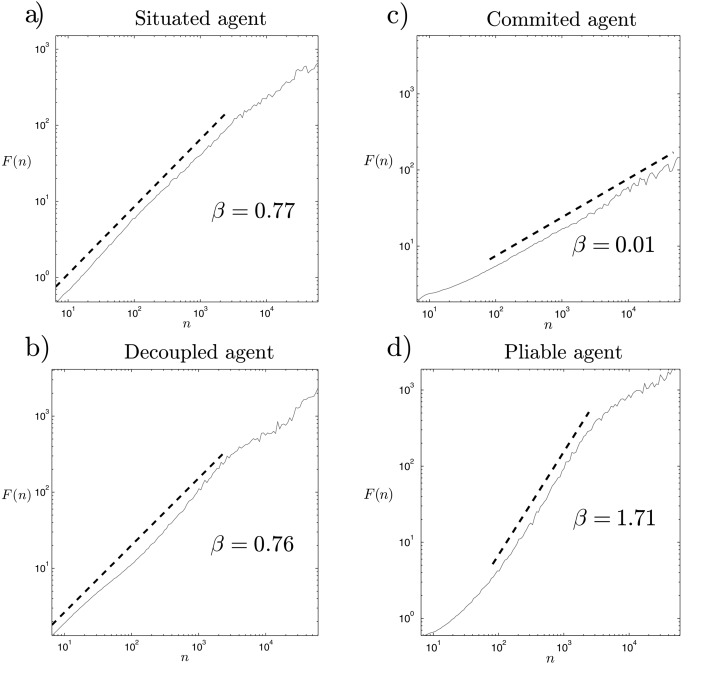
Results of the detrended fluctuation analysis. We observe for the situated and decoupled agents, a) and b) that the DFA analysis shows a scaling close to 1/*f* noise. The agent without synaptic plasticity displays either c) white noise for committed agents or d) brown noise for pliable agents.

If we take different series of the variable Φ for the situated agent and apply the DFA algorithm to them, we find that the signal presents linear relationships on a logarithmic scale from 1*s* up to around 10^3.5^ *s* (Fig. 6). If we compute the *β* coefficient we find that it is quite close to pink noise. We can run the simulation several times for different initial values and we always find quite similar results, with *β* around 0.77. This result suggests that the process of the emergence of complex patterns analyzed above is generated by SOC.

For the case without synaptic plasticity, we obtain different results depending on the ‘frozen’ weight configuration. Specifically, we obtain quite different results for two different kinds of agent: 1) *committed* agents that always go to the same light, and 2) *pliable* agents that chose one light or another depending on their initial positions. We can generalize the results in two different kinds of DFA results (Fig. 6.c and 6.d). The committed agents (as in the cases of Figs. 3.b and 3.c) display the signature of Φ with a characteristic white noise structure (*β* = 0.01), where the absence of plasticity leads to a situation where the agent has no ‘memory’ of its previous trajectories. Pliable agents, which are sensitive to both lights (Fig. 3.d), present a narrower fractal spectrum and display a scaling with a value of the *β* coefficient around 1.71, characteristic of brown noise signals. These agents present a much stronger ‘memory’ than pink noise signals, showing short-term predictabilities in their activity. In both cases, when synaptic plasticity is removed, the system clearly losses its ability to critically self-organize.

Finally, if we apply DFA to the series generated from the decoupled agent, we obtain something that appears to be similar to the results for the situated agent (Fig. 6.b). However, the slope no longer appears to be so straight. Although it resembles a 1/*f* pattern, in DFT analysis we show how in this case (as opposed to the case of a situated agent) DFA results can be misleading and need to be validated with further analysis in order to reliably characterize SOC.

#### Discrete Fourier Transform

**Fig 7:**
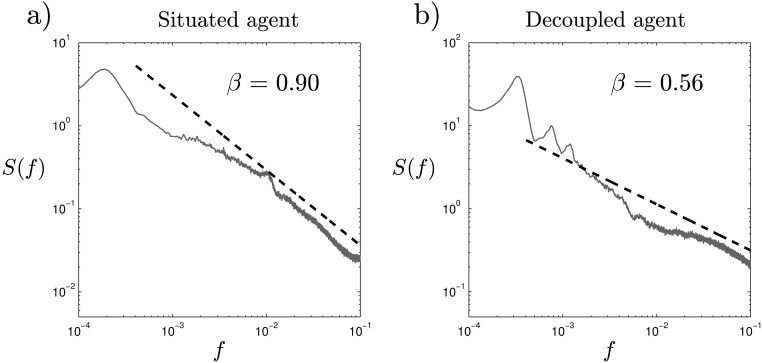
Result of the discrete Fourier transform. We observe the differences between the situated and decoupled agent and how deviations from linearity in the decoupled agent are much more noticeable in DFT analysis than in DFA analysis.

We have seen that plasticity plays an important role in the generation of SOC dynamics, but that the situatedness of the agent does not display significant differences according to DFA exponents (despite the significant differences shown by the analysis of neurodynamic patterns). To further test this result, we have computed an averaged DFT spectrum. Since DFT results are noisier than DFA for the 1000 trials series of Φ, we average the resulting DFT over 100 independent runs (each one consisting of 1000 trials) with random initial conditions. The averaged DFT shows us the difference between the situated and decoupled spectrum with greater precision (Fig. 7). Since in DFA we have to look for slopes between 0.5 and 1.5 while in DFT they range between 0 and 2, we can better appreciate the difference between both signals. For the situated signal, we find a region with a fractal scaling for frequencies between 10^−1^ *Hz* and 10^−3.5^ *Hz*, that is, very close to pink noise (*β* = 0.90) which is only distorted for lower frequencies. Moreover, if we reduce the range in which we compute the fractal scaling for DFA analysis (see Fig. 6.a) for a smaller temporal range of *n* = [10^1^ *s*, 10^2.5^ *s*]) we find that the exponent *β* rises up to 0.88.

Moreover, for the decoupled case (Fig. 7.b), we find that what appeared to be a slight curved line in DFA analysis is much more noticeable in DFT analysis. The signal obtained no longer displays a clear linear relation and the slope of the linear fitting is much lower (*β* = 0.47). Thus, DFT analysis suggests that the decoupled agent does not display SOC. Nevertheless, it is yet to be confirmed that the decoupled system does not present the kind of nonlinear interaction-dominated threshold dynamics necessary for SOC. We now analyze how 1/*f* patterns only arise in a robust manner for the situated case.

#### Interaction-Dominant Dynamics and Multifractality

The constructive definition of SOC [11] emphasizes that SOC systems are interaction-dominated threshold systems driven by inherently nonlinear coordinative mechanisms. Ihlen and Vereijken have proposed that 1/*f* noise is neither necessary nor sufficient evidence of interaction-dominant dynamics and that multifractal analysis is a quantitative framework suitable for the analysis of interaction-dominant behaviour [35]. The multifractal spectrum quantitatively defines the presence of multiplicative interactions between temporal scales that are responsible for the emergence of intermittent, emergent or coherent periods of large fluctuations within the response series of a system. In [35] it is shown how critical and super-critical neural network models display a multifractal structure, whereas subcritical networks (where multiplicative interactions across temporal scales do not take place) show monofractal structures.

We use the Continuous Wavelet Transformation (CWT) algorithm to calculate the multifractal spectrum. The CWT uses a Morlet waveform to decompose the response series into a continuous range of temporal scales [51]. This decomposition allows us not only to analyze the scaling of the variance of a signal (as in DFA) but also the entire probability density function defined by all *q*-order statistical moments through a series of local exponents *h* associated with each *q*. The width of the multifractal spectrum Δ*h* = *h*_*max*_ − *h*_*min*_ defines the amplitude difference between the variability in the intermittent and laminar periods of the response series, quantifying the influence of the multiplicative interaction, or coordinations, between the multiple time scales of the response series.

We have computed both the *β* coefficient from DFA analysis and the multifractal spectrum of the variable Φ for 25 different runs of the four types of agents (situated, committed, pliable and decoupled). This time (since we already know where the interesting parts of the spectrum lie) we restrict DFA analysis to the interval *n* = [10^1^ *s*, 10^2.5^ *s*] for the situated and decoupled agents, and the interval of *n* = [10^2^ *s*, 10^3.5^ *s*] for the pliable and committed agents. Due to computational reasons, for the multifractal analysis we have decimated the time series by a factor of 10, although similar results were found for individual runs without decimation. As in [35] we have only computed the lower half of the spectrum, using a lower bound of *q* > 0, since the CWT yield unstable estimations of negative moments. The upper bound was set to *q* = 10.

**Fig 8:**
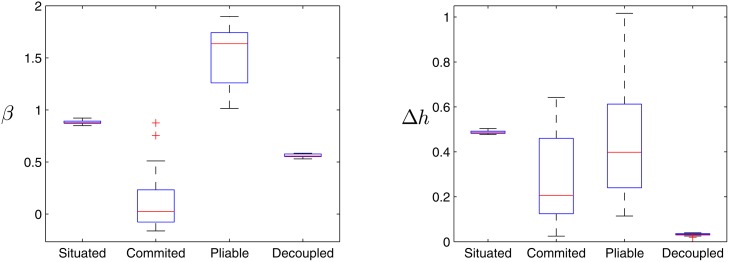
Fractal exponents and width of the multifractal spectrum of the four types of agents. Only situated agents present both a pink noise exponent and a wide multifractal exponent. Decoupled agents appear to present a 1/*f* exponent but this is a misleading sign of SOC because they show no multifractality.

The results of the fractal and multifractal analysis are shown in Fig. 8. We can see how: a) the situated agent shows a pink noise exponent (mean *β* of 0.88) and a broad multifractal spectrum (mean Δ*h* of 0.49) with a very high consistency between trials; b) the committed agent without synaptic plasticity displays a white noise exponent (mean *β* of 0.02) and a variable amplitude of the multifractal spectrum, ranging between 0.02 and 0.64, with a mean of 0.21; c) the pliable agent presents a brown noise exponent (mean *β* of 1.65) and a variable multifractal spectrum between 0.11 and 1.02 and with 0.40 mean. This may show how different configurations of the network weights leads to different kinds of dynamics. Note that supercritical dynamics have been shown to display broader multifractal spectra than critical dynamics [35]). Finally, d) the decoupled agent displays a pink noise exponent (mean *β* of 0.56) and quite a narrow monofractal spectrum (with Δ*h* around 0.03), suggesting that the system is not driven by interaction-dominant dynamics and presents a much more limited coordination between timescales.

This confirms our suspicion about the decoupled agent not being a critically self-organized system, since the emergent dynamics do not arise from a process of multiplicative interactions between components, but from a component-dominant dynamics.

#### Response to Parametrical Changes in the Agent’s Configuration

As we have seen, agents with synaptic plasticity and active coupling to the environment display both pink noise and multifractal patterns, as opposed to agents without synaptic plasticity or agents without sensorimotor coupling. However, decoupled agents present a signature which looks similar to a 1/*f* pattern even if they have a monofractal spectrum, and this may be misleading for characterizing them as SOC systems. Typically, a system is defined as critically self-organized when it displays critical dynamics without any fine tuning by an external driving influence. In order to test the robustness of the agents above by measuring 1/*f* and multifractal patterns when the model’s parameters are changed, we have run a series of simulations of the situated and decoupled agents which display different parametrical changes in the configuration of the model. The simulated agents are as follows:

1. A normally functioning agent
2. An agent presenting a random permutation of the values of *ϕ*_0_.
3. An agent presenting a random permutation of the values of *ω*.
4. An agent presenting a random permutation of the values of *η*.
5. An agent presenting an alteration of the parameter *α*. The value of *α* is multiplied by a constant *k*_*α*_ = 2^*r*^, where *r* is a random number distributed uniformly in the interval [−1, 1].
6. An agent presenting an alteration of the value of *H*_1_. The value of *H*_1_ is multiplied by a constant *k*_*H*_1__ = 2^*r*^, where *r* is a random number distributed uniformly in the interval [−1, 1].

**Fig 9:**
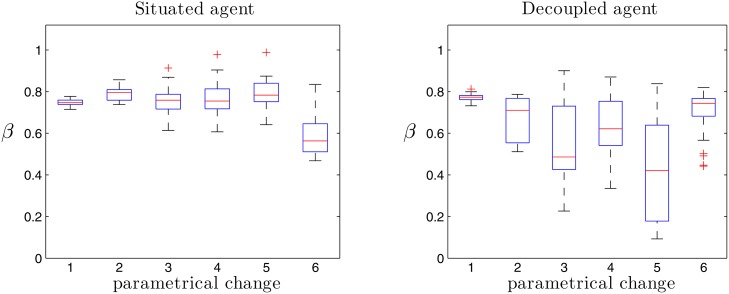
Fractal analysis of the patterns emerging under different parametrical changes. Whereas 1/*f* patterns are robust for the situated agents the fractal exponents are drastically reduced for the decoupled agent.

For each condition of the list above we have simulated the behaviour of both the situated and the decoupled agents for 25 runs during 125000*s*, with the same configuration as the other simulations described in this section. Surprisingly, all the situated agents with parametrical changes were able to behave in a phototactic manner displaying transient preferences for the two lights. For each simulation we performed DFA and multifractal analysis for the variable Φ over the range *n* = [10^1^ *s*, 10^3.5^ *s*]. The results can be seen in Fig. 9.

We observe how pink noise patterns are quite robust for the situated agents, which always display fractal exponents close to 1 with small variability. The only situation in which the fractal exponent becomes slightly lower is in the case of the alteration of the threshold *H*_1_ that triggers plasticity in the neural controller. This suggests that nonlinear thresholds dynamics is a crucial element for the generation of SOC.

On the other hand, fractal exponents for the decoupled agent are dramatically reduced when parametrical changes are applied. Thus, we cannot attribute a genuine SOC for the decoupled agents, since small changes in the parameter of the agents destroy the criticality in the system. This suggest that in this case criticality is produced by tuning of the oscillator network parameters, so pink noise-like exponents are more likely to be result a right set of parameters from the genetic algorithm.

## Discussion

Self-organized criticality refers to the phenomenon whereby a dissipative dynamical system with many degrees of freedom operates near a configuration of minimal stability, i.e., a critical configuration, and does so without any fine tuning by an external driving influence. For SOC to emerge, the dynamics of a system must be dominated by mutual interaction of the many degrees of freedom comprising the system, interaction-dominant dynamics being a necessary condition for the emergence of SOC. Also, SOC systems usually display robust 1/*f* patterns without fine parameter tuning, being resistant to alterations in the system’s parameters. In this work we have proposed the use of scale-free exponents (fractal and spectral) and multifractal exponents as indicators of SOC and IDD respectively, and we have proposed a methodology for accurately characterizing SOC ruling out false positives when characterizing 1/*f* patterns.

We have presented an agent controlled by a plastic neural controller which performs a behavioural preference task, choosing alternatively between two lights and generating stable but transient preferences to one of the lights. The agent displays an adaptive and flexible behaviour, being able to overcome changes in its parametrical configuration. It also shows stable behavioural patterns of light preference. Scale-free exponents and multifractal analysis show that the agent displays SOC in the dynamics of its neural controller (without this controller ever being explicitly selected to display SOC). Moreover, SOC dynamics are stable in the face of parametrical changes. The only situations in which SOC is no longer displayed arise when the agent loses either its synaptic plasticity or its sensorimotor coupling with the world.

Research on 1/*f* patterns and SOC in cognition has been typically divided between advocates of two apparently irreconcilable views: nomothetic perspectives on 1/*f* noise on the one hand promoting general explanations of 1/*f* noise [8, 20], and mechanistic perspectives on 1/*f* noise on the other, focused on modeling concrete processes underlying particular phenomena or calling for physiologically detailed mechanistic models [16, 36]. Some of the latter accuse the former of proceeding ‘by identifying a mysterious phenomenon (i.e., 1/*f* noise) and explaining this phenomenon by verbal reference to a series of other mysterious phenomena (e.g., SOC), without ever making contact with latent cognitive processes’ [33, p. 91]. It is the tendency to ignore specific mechanisms that demands attention, since the alleged lack of explanatory power of nomothetic perspectives (making verbal references to other mysterious phenomena) is motivated by a lack of reference to constituent components of the explanation. The very proponents of nomothetic or so called ‘dynamical explanations’ state that such explanations ‘do not propose a causal mechanism that is shown to produce the phenomenon in question’ [52, p. 432] and often rely on higher level principles where ‘the change over time in a set of magnitudes in the world can be captured by a set of differential equations’. Appeals to SOC, or interaction-dominant systems, for *explaining* 1/*f* scaling are argued to fall within this category of nomothetic explanations devoid, according to Wagenmakers, of genuine explanatory potential.

We consider that the terms *dynamical* or *complex systems* to characterize a type of model or explanation need not to be equated with nomothetic explanations. Dynamical systems theory, very much like computational theory or algebra, can be used to build either mechanistic or nomothetic explanations. Moreover, following Kaplan and Bechtel’s call for complementarity between nomothetic and mechanistic explanations [53] we believe that much is to be learned by attempting to connect and explore the relationship between behaviour generating mechanisms (at different levels of abstraction) and the regularities displayed by such mechanisms in operation. It is here where conceptual models [40, 54, 55] can be useful. Conceptual models, such as the robotic model presented here, need not model specific target systems (i.e. a natural system, its behaviour and its physiological processes) but might bring into the model abstract mechanisms, general principles or generic processes, in order to study the relationship between different levels of explanation (e.g. between mechanistic and nomothetic explanations), between different types of phenomena (e.g. between 1/*f* noise and SOC principles) or between different measurements and the principles or properties they capture (e.g. fractal and multifractal structures and interaction-dominant dynamics) providing valuable insights for future empirical experimentation and modelling.

Note that the model presented here, being abstract and not empirically driven, does not prove that the properties just mentioned apply to natural behaving organisms, but rather that it could be the case. In this sense the model works as a proof of concept that can inform and drive future experiments operating as what Chemero calls ‘a guide to discovery’ [55, 56]. Thanks to our model we can hypothesize that, at least for some cases, SOC and 1/*f* patterns in behaving systems *might* be the result of: a) non-linear interaction dynamics capable of generating stable collective patterns, b) internal plastic mechanisms that allow self-sustained criticality through a continuous modulation of sensorimotor flows, c) strong sensorimotor coupling with the environment that induces transient metastable regimes and, d) a small number of behaviour generating components or variables. The last three elements are relatively novel, in relation to previous models of SOC, and deserve detailed discussion.

Previous works have highlighted the importance of synaptic plasticity for the emergence of a critical state [16, 57]. The ability to sequentially recruit neurons or neural areas into different clusters of coordinated oscillators seems crucial for the brain to self-regulate its activity, resting always at the brink of criticality. In our model, when internal plastic mechanisms are not present the agent seems to get stuck in either: a) a state that is closer to a subcritical state (white noise, narrow multifractal spectrum) in which stimuli are not sufficient to change the agent’s preference (‘committed’ agents) or b) what resembles a supercritical state (brown noise, wide multifractal spectrum) in which the agent just responds immediately to any stimulus from any of the lights (‘pliable’ agents). In this sense, plastic mechanisms seem to mediate between internally driven and externally driving dynamics, allowing a conjunction of internal and external influences that produce a state of SOC. Experimental studies have shown that 1/*f* scaling appears more clearly in constrained motor tasks as participants become trained [58], suggesting that plastic changes may drive the system’s performance towards a state of SOC. Moreover, enhancing or decreasing sources of constraint in the voluntary control of an agent will shift scaling exponents from pink noise towards brown or white noise respectively [59]. Similar mechanisms may operate among the different kinds of agents presented here.

On the other hand, when sensorimotor coupling is not present, although the fractal spectrum of the signal has some similarities with the 1/*f* spectrum of the coupled agent, the multifractal analysis displays a monofractal spectrum, indicating that the interaction-dominant dynamics of the system has been lost, and now the system is driven by the sum of the dynamics of individual components. An analysis of the behaviour of the system under parametrical alteration confirms that the decoupled agent does not present robust 1/*f* patterns. Thus, we find that in our model a strong sensorimotor coupling is essential for coordinating the dynamics of the components of an agent into a critically self-organized dynamic unity, thus extending the notion of interaction-dominant dynamics to the sensorimotor loop itself (and not just to the internal interaction between components: e.g. neurons or brain regions).

Another interesting aspect of the model is that whereas it is typically considered that one of the features of a SOC system is having a large number of units (neurons), the model presented here is driven by only three oscillators with their respective plastic synapses. We have shown that a network of just three oscillators is capable of generating SOC provided that plastic mechanisms are in place and the system is situated in a sensorimotor environment. That is, as opposed to most models of SOC, a huge number of components need not be necessary to generate 1/*f* noise. What counts as a component, however, is subject to debate. There are two ways in which oscillators can be interpreted in our model: they can be taken to represent single neurons or specific neural mechanisms or, alternatively (this is the interpretation we favour) as mesoscopic structures (e.g. brain regions). The implication of the second interpretation is that SOC need not be captured at a localized microscopic level of behaviour generating mechanims (e.g. individual neurons), but could perfectly be described as resulting from meso or macroscopic regularities that might result from different local mechanisms.

Other features often associated with SOC, such as stochastic variation of internal parameters, or the ability to store information in spatial patterns, appear not to be present in the model. On the contrary, as opposed to most models of SOC, our agent is strongly engaged in a rich sensorimotor interaction with an environment that affords specific sensorimotor contingencies for an embodied agent. We have proved that sensorimotor coupling is a crucial aspect for our model to display SOC, and we show that it is not the neural controller on its own that presents critical self-organization but the extended brain-body-environment system as a whole. Although 1/*f* noise has been found to appear spontaneously out of interconnected neuronal networks ([15–16, 37]), in some cases the very contribution of the sensorimotor loop might be crucial for generating genuine 1/*f*. This calls for an integrated agent-environment modelling of cognitive processes. Our model shows, that among interaction-dominant processes, sensorimotor interaction, and not only (or perhaps not even) internal neuronal-muscular-etc processes, could be the crucial dominating interaction in the production of 1/*f* noise.

This idea provides interesting insights into the notion of a dynamic core as a necessary hub for agencial and conscious processes. Usually a dynamic core is circumscribed to distributed clusters of neurons intensely interacting with each other within the brain. We find appealing the notion of a dynamic core composed not only of coupled neural dynamics, but extended to coupled neural and environmental reentrant processes: a *sensorimotor dynamic core*. Edelman and Tononi stress that a dynamic core is a process, not a thing or a place, and is defined in terms of neural interactions, rather than in terms of specific neural locations, connectivity or activity [25]. Thus, there is no apparent reason to limit the definition of a dynamic core to brain-bound events. Our model shows instead, that the dynamic core might well be extended, cutting across brain-body-world divisions. This view fits the ‘radical embodiment’ framework proposed by Thompson and Varela [60], in which specific neuronal assemblies underly the operation of cognitive acts, depending crucially on the manner in which brain dynamics are embedded in the somatic and environmental context of the agent’s life. Our model thus provides a generative illustration of the generic intuition outlined by Silberstein and Chemero.

The model presented here has tried to highlight the importance of taking into account a framework that includes the coupled dynamics of the brain-body-environment system as a whole. We have shown how a minimal behavioural model can display self-organized criticality when we allow strong interactions between plastic neural mechanisms and sensorimotor processes. We have tested different measurements of SOC in non-linear sensorimotor dynamics, we have carried out a systematic comparison of such methods, together with experimental procedures to accurately test the presence of genuine SOC, and we have provided a simulated proof of concept of how these measurements can be systematically applied to a simple cognitive agent. By clarifying the relationship between different types o properties and processes capable of generating SOC and 1/*f* noise in cognitive systems we hope to have opened the way for more accurate models and experiments that may shed light to the widespread presence of 1/*f* noise in natural agents.

## Supporting Information

S1 Video Video of the behaviour of a situated agent. Top left: position of the agent and the two lights. Top rigth: phase relationships of the oscillators and levels of plasticity. Bottom left: final distance to each light at every trial. Bottom right: activation of the sensors, motors and connection weights.
(MP4)
